# Rheological Response, Strength Development and Conditional Age-Progressive Strength Updating of Cement Kiln Dust-Modified Cemented Tailings Backfill

**DOI:** 10.3390/ma19183934

**Published:** 2026-09-16

**Authors:** Chong Chen, Wenquan Duan, Junsheng Ma, Qian Wen, Qingchun Hu

**Affiliations:** 1 Norin Mining Limited, Beijing 100035, China; chenchongcsu@gmail.com (C.C.);; 2 School of Resources and Safety Engineering, Central South University, Changsha 410083, China; 3 Hunan Industry and Trade Vocational School, Changsha 410005, China

**Keywords:** cement kiln dust, cemented tailings backfill, chemical admixtures, rheological properties, compressive strength, age-progressive prediction model

## Abstract

Cement kiln dust (CKD) can partially replace Portland cement in cemented tailings backfill, but its application must satisfy both pipeline transportability and mechanical strength requirements. This study aims to evaluate the rheological and mechanical evolution of a low-carbon CKD-modified backfill to optimize its transport–strength trade-off. This study introduced a binder containing 10% CKD and 90% ordinary Portland cement at a binder-to-tailings ratio of 1:6. Thirteen single-factor mixtures were prepared to examine silica fume (0–7% of binder), sodium silicate (0–4%), polycarboxylate powder (0–0.40%), and solids mass concentration (72–74%). The Bingham yield stress and plastic viscosity were determined in the fresh state, and unconfined compressive strength (UCS) was determined after 3, 7, 28, and 56 d. Increasing polycarboxylate dosage strongly reduced yield stress, whereas raising solids concentration from 72% to 74% increased yield stress from 5.82 to 58.83 Pa and plastic viscosity from 0.0953 to 0.2889 Pa·s. Silica fume increased 56 d UCS from 1.39 to 2.28 MPa, while sodium silicate mainly improved early-age strength and produced non-monotonic later-age responses. At 74% solids, the 56 d UCS reached 2.54 MPa, accompanied by substantially higher rheological resistance. This study introduces a path-dependent, age-progressive framework to improve backfill strength prediction accuracy. A conditional age-progressive regression model was developed using min–max normalized mix variables and the measured UCS at the preceding age. In-sample R2 values were 0.9697–0.9805 for 7–56 d; leave-one-out validation with measured prior-age UCS gave R2 values of 0.7951–0.8198. The model should therefore be interpreted as a field-updating tool after early-age testing rather than as an independently validated design-only predictor. The results quantify the transport-strength trade-off within the investigated materials and dosage ranges.

## 1. Introduction

Mining and mineral processing operations generate massive volumes of tailings and other industrial residues. Inadequate storage consumes land resources, contaminates water systems, and increases the risks and consequences of tailings-storage-facility failure [1,2,3]. Cemented paste backfill (CPB) is a critical engineering methodology that facilitates in situ tailings disposal, stabilizes underground stope, and minimizes the surface environmental footprint [4,5]. However, conventional CPB practices depend heavily on ordinary Portland cement (OPC), a binder characterized by high energy consumption and significant carbon emissions [6,7]. This reliance imposes restrictive operational expenditures on mining projects in developing regions. Consequently, developing an alternative, low-carbon, and solid-waste-modified binder matrix has become an urgent scientific and engineering priority.

Cement kiln dust (CKD) is collected from kiln exhaust-gas treatment systems during clinker production [8]. CKD is characterized by an exceptionally high calcium oxide CaO content, demonstrating strong potential as an alkaline activator and a supplementary cementitious material [9,10]. When integrated into a composite binder matrix, CKD quickly hydrolyses to release substantial concentrations of hydroxyl ions (OH^−^), elevating the pore solution pH and breaking the structural bonds within amorphous mineral phases [11,12]. Despite these chemical advantages, solid waste-based backfill matrices frequently experience a delicate trade-off between rheological transportability and structural strength evolution, particularly in ultra-high concentration pipelines [13,14,15]. To optimize this balance, chemical admixtures like silica fume, sodium silicate, and polycarboxylate superplasticizers are often introduced into the paste. Silica fume optimizes the physical particle packing density while supplying highly active amorphous silicon dioxide (SiO_2_) for mid-to-late pozzolanic reaction stages [16]. Sodium silicate induces rapid early-stage calcium silicate hydrate (C-S-H) gel crystallization [17,18], and polycarboxylate molecules generate steric hindrance and electrostatic repulsion to disperse fine particles [19]. The existing literature focuses primarily on single-admixture mechanisms within pure OPC systems; consequently, the individual mechanistic effects of these admixtures in CKD-tailings multiphase suspensions remain under-explored. Establishing these isolated baseline behaviors is a critical prerequisite for the future development of multi-admixture optimization protocols.

Accurately predicting the lifecycle strength evolution of backfill is essential for determining stope cycle times and ensuring surrounding rock mass stability. Previous studies typically employed empirical multivariable regression or black-box machine learning algorithms to establish mathematical correlations between initial mix components and compressive strength outputs [20,21]. Cementitious hydration is a continuous non-equilibrium thermodynamic process in which the evolution of mechanical properties is inherently time-dependent [22]. Conventional prediction models typically treat strengths measured at different curing ages as independent static outputs, thereby neglecting the temporal continuity of hydration, and overlooking the structural path dependency of material development. Therefore, incorporating a sequential succession variable that reflects this structural path-dependency is critical for improving strength prediction from mathematical curve-fitting to a physically grounded evolutionary representation.

This study investigates a low-carbon cementitious system consisting of 10% cement kiln dust (CKD) and 90% ordinary Portland cement (OPC) blended with high-silica total tailings. Based on a single-factor experimental design involving 13 experimental groups, the effects of silica fume, sodium silicate, polycarboxylate powder and solid fraction on the rheological behavior and multi-age unconfined compressive strength (UCS) development are systematically evaluated. Despite the recognized potential of CKD, a critical research gap remains in quantifying the trade-off between the transportability and mechanical strength in complex CKD–tailings suspensions. In addition, the existing prediction approaches of backfill rarely consider the time-dependent development of structural continuity. Therefore, this study aims to systematically evaluate and optimize the rheological transportability and strength development of a low-carbon backfill matrix. This study first quantifies the individual effects, such as silica fume, sodium silicate, polycarboxylate powder, and solid fraction on Bingham rheological parameters and multi-age unconfined compressive strength (UCS) and then proposes a predictive mathematical model involving initial mix variables to life-cycle mechanical performance. The primary novelty of this study is the development of a conditional age-progressive prediction framework that captures the evolution of mechanical performance as the backfill structure develops with curing time. By incorporating the strength state at the preceding curing age as an integrated descriptor of the evolving microstructure, the proposed model considers the temporal continuity of cement hydration, reduces the influence of experimental noise through state inheritance, and significantly improves predictive accuracy. The proposed method can provide both mechanistic insights into the evolution of low-carbon cemented backfill and a grounded framework for the industrial utilization of CKD in sustainable mine backfill applications.

## 2. Materials Properties and Experimental Methodology

### 2.1. Raw Materials and Microstructural Geochemical Characterization

**Figure 1:**
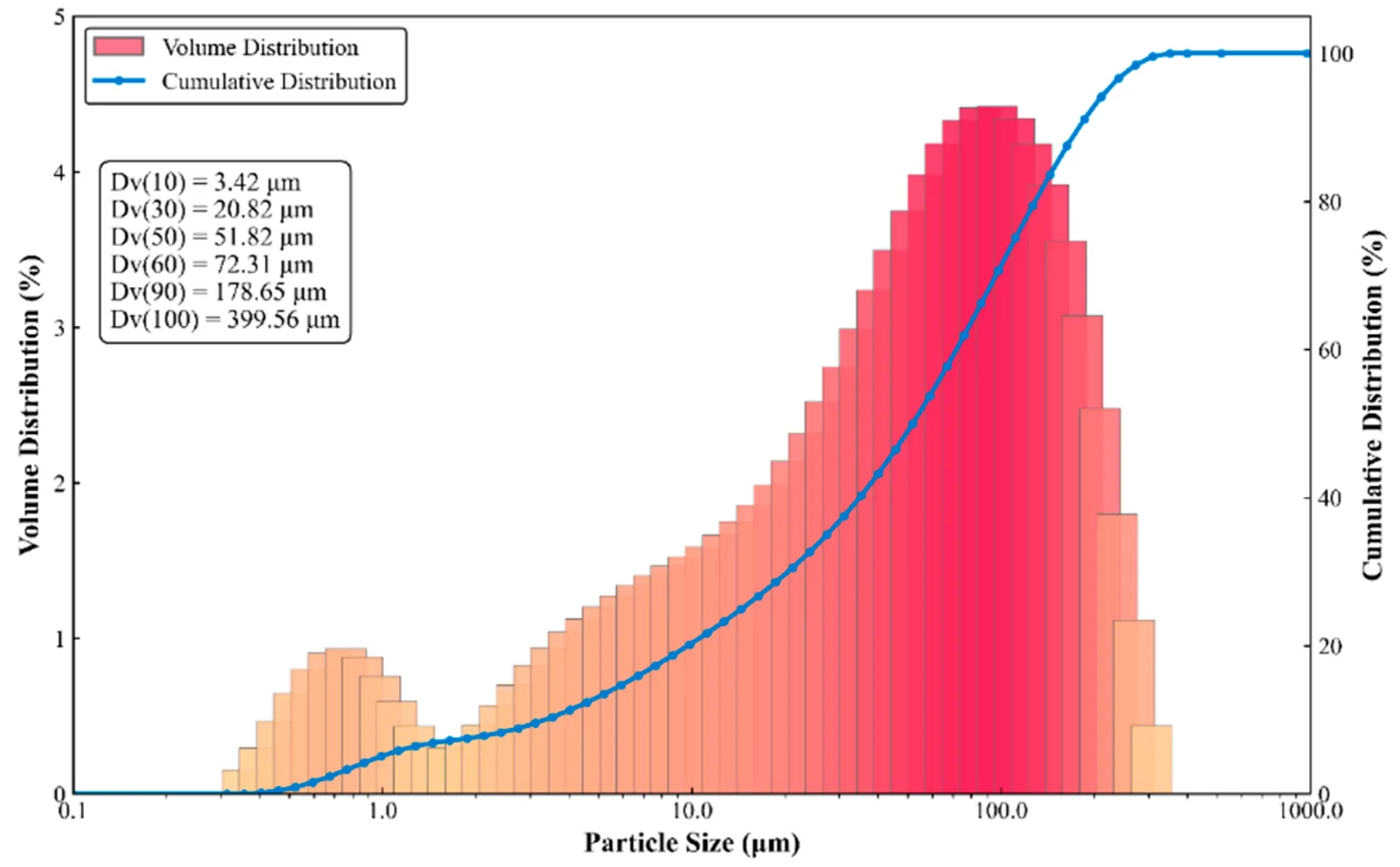
Particle size distribution of tailings.

The total tailings utilized as the structural aggregate were gathered from the processing plant of an underground mine in Africa. Physical property evaluations indicate that the aggregate has a true density of 2.699 g/cm^3^, a loose bulk density of 1.251 g/cm^3^, and a corresponding initial porosity of 54%. Particle size distribution analysis, conducted using a Malvern Mastersizer 3000 (Malvern Panalytical Ltd., Malvern, UK), yielded the following key grain size parameters: d_10_ = 2.003 μm, d_30_ = 11.201 μm, d_50_ = 29.675 μm, d_60_ = 51.823 μm and d_90_ = 183.916 μm, as shown in Figure 1. The chemical characterization of the total tailings is listed in Table 1, revealing that SiO_2_ is the dominant structural component, accounting for 46.45% of the mass fraction, which classifies it as a typical siliceous aggregate. Other primary mineral constituents include CaO (12.98%) and MgO (11.57%), whereas the weight percentages of Al_2_O_3_, Fe_2_O_3_, Na_2_O, and K_2_O remain below 2.5%.

The multi-phase cementitious binder system is composed of ordinary Portland cement (OPC, grade 42.5 N) blended with cement kiln dust (CKD). Table 2 shows that the main components of OPC are CaO and SiO_2_. The geochemical composition analysis in Table 3 indicates that the core compound of the CKD is CaO at an exceptionally high concentration of 58.10%, aligning with the high-calcium signature of conventional clinker materials. However, its cumulative mass fraction of acidic oxides (SiO2, Al_2_O_3_, Fe_2_O_3_) is only about 14%, thereby possessing a relatively weak innate hydraulic reactivity foundation.

### 2.2. Multi-Phase Mix Formulation Matrix Design

To systematically evaluate the individual effects of each chemical admixture and the solid mass concentration on the rheological and mechanical properties of the backfill, thirteen mixtures (S1–S13) were prepared using a single-factor screening design (Table 4). The baseline composite binder consists of 10% CKD and 90% OPC by weight. The binder-to-aggregate (cement-to-tailing) ratio was fixed uniformly at 1:6 across all test specimens. The slurry mass concentrations varied between 72% and 74%. All chemical admixture dosages were computed as a weight percentage relative to the total mass of the binder system. The selected operational ranges were determined through preliminary single-factor trials. The selected operational ranges for the chemical additives were defined as follows: silica fume (SiO_2_ ≥ 85%) at 0–7%, sodium silicate powder (Na_2_O·nSiO_2_, modulus n = 2.4) at 1–4%, and polycarboxylate powder (solid content ≥ 95%) at 0.10–0.40%. When exploring the variations of a specific admixture under the baseline concentration (72%), all other chemical additives were strictly maintained at 0%. The comprehensive multivariable mix formulations are cataloged in Table 4 (C denotes slurry concentration; M denotes silica fume; S denotes sodium silicate; P denotes polycarboxylate powder).

### 2.3. Rheological and Mechanical Testing Setup

**Figure 2:**
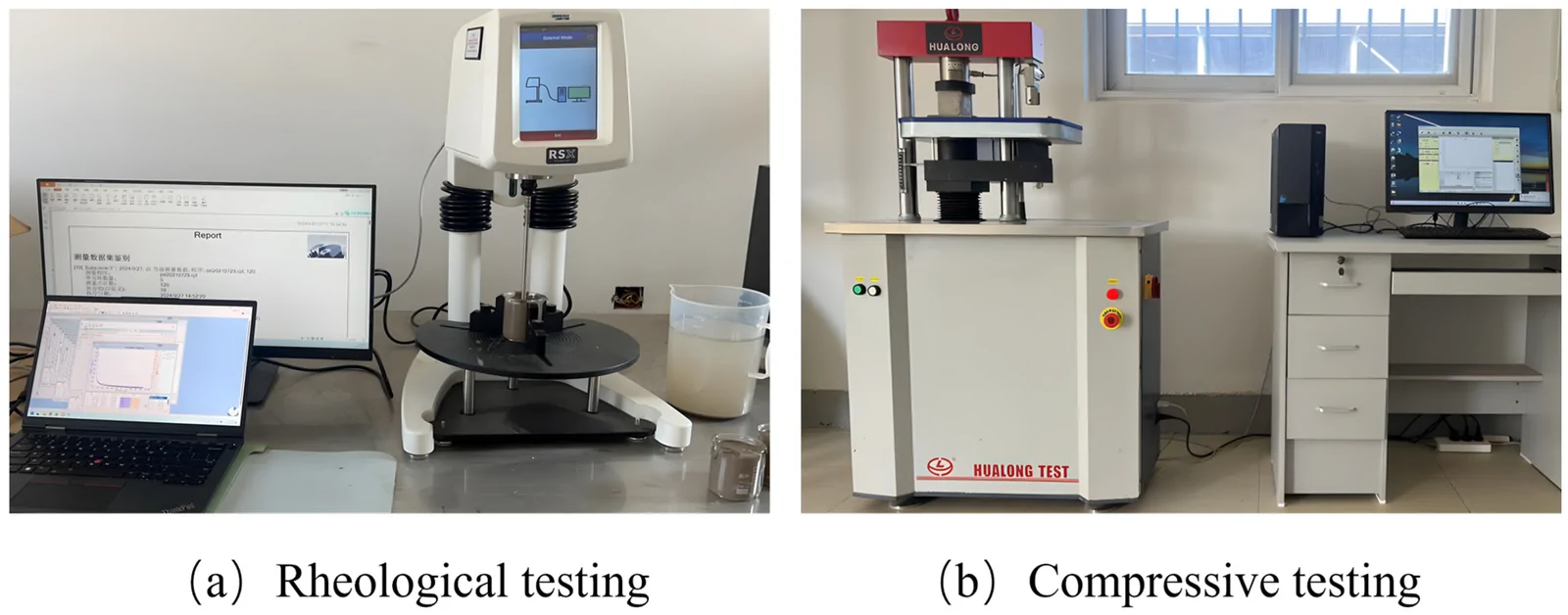
Experimental setups for rheological and compression tests.

The tailings were oven-dried and mechanically disaggregated before mixing. According to the proportions specified in the experimental matrix, the dry solid components (tailings, OPC, CKD, and any solid chemical admixtures) were first dry-mixed in a standard laboratory mixer at a low speed for 3 min to ensure uniform dispersion. The designated amount of mixing water was then gradually introduced, followed by continuous high-speed mixing for an additional 6 min to achieve a completely homogenous slurry and eliminate particle agglomeration. The macro-rheological properties, specifically the structural yield stress and plastic viscosity, were quantified immediately using a Brookfield RST-SST soft solid rheometer (AMETEK Brookfield, Middleboro, MA, USA), shown in Figure 2a, in which the maximum shear rate for the rheological test was set at 120 s^−1^, the shear rate gradually increased from 0 to 120 s^−1,^ and the rheological test time for each slurry was 120 s. The measurement protocol included a 30 s low-shear phase to homogenize the sample, followed by a 60 s rest period to allow for structural recovery. The testing procedure utilized a controlled shear rate ramp, and the resulting flow curve data were fitted to the Bingham fluid model (shown in Equation (1)) to derive the fundamental yield stress and plastic viscosity parameters. Bingham model is a special case of the Herschel–Bulkley model when the flow index equals to 1. During the data evaluation, the analytical software simultaneously compared the fitting results of the linear Bingham model and the non-linear Herschel–Bulkley model. The regression diagnostics revealed that the flow index for the tested slurries was consistently close to 1.0 within the specified shear-rate range (0–120 s^−1^); ultimately, the Bingham model was selected as the optimal mathematical fit. To ensure data reproducibility and eliminate operational anomalies, each rheological measurement was performed three times, and the mathematical average was reported. To explicitly account for experimental variability, the results are reported as the mathematical average ± standard deviation (SD).

Subsequently, the fresh mixtures were cast into standard triple-gang plastic molds with dimensions of 7.07 cm × 7.07 cm × 7.07 cm, which is the explicitly specified dimension for heterogeneous cemented tailings backfill. To eliminate entrapped air and ensure structural density, the filled molds were compacted on the vibrating table for 60 s. The cast specimens were allowed to set for 24 h before demolding. The cubes were transferred to a standard curing chamber (temperature: 20 ± 2 °C; relative humidity: ≥95%; specimen spacing: ~10 mm). After a 24 h curing period, the molds were removed, and the specimens were grouped and labeled accordingly. The specimens were then returned to the curing box and maintained under controlled conditions until they reached the designated curing ages. The uniaxial compressive strength (UCS) evaluations were executed by the compression machine (illustrated in Figure 2b) at designated curing lifecycle milestones of 3 d, 7 d, 28 d and 56 d. An extended 56-day curing period was evaluated because the cemented backfill contains a low binder dosage and a high water-to-binder ratio, and the supplementary cementitious materials (such as silica fume) and solid waste activators (such as CKD) rely on secondary pozzolanic reactions. The specimens were loaded in displacement control at 0.5 mm/min, while load-displacement data were continuously captured through the post-yield phase to 20% of peak load. For each distinct lifecycle milestone within every group, three independent companion specimens were tested, and the final mechanical strength value was defined as the statistical average.

## 3. Shear Rheological Kinetics and Fluidic Drag-Reduction Mechanisms of Backfill Slurries

**Figure 3:**
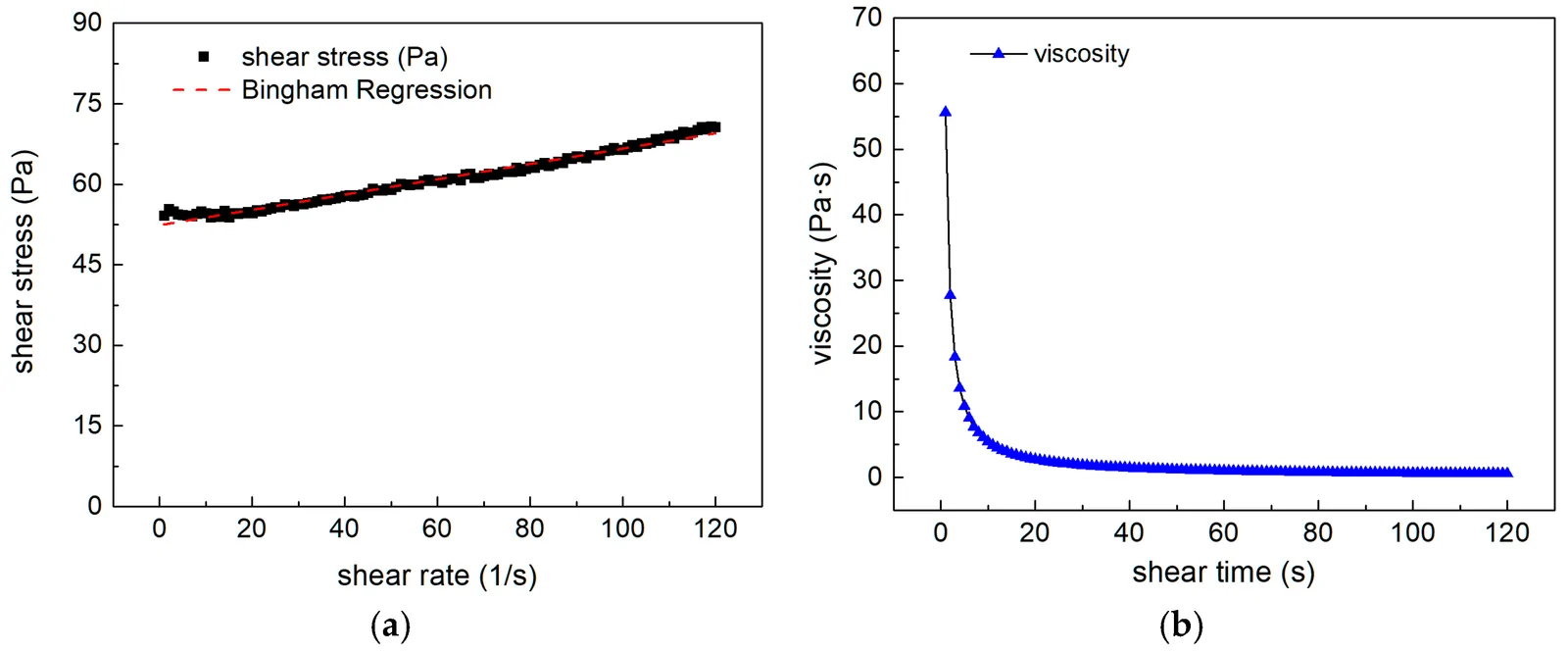
Rheological curves of backfill slurry. (**a**) Correlation between shear stress and shear rate of slurry; (**b**) correlation between apparent viscosity and shear time of slurry.

The macroscopic transportability of CKD-modified cemented tailings backfill (CCTB) is fundamentally dictated by its fresh-state rheological properties. The evolution of yield stress (τ_0_) and plastic viscosity (μ_p_) under diverse multi-phase combinations exhibits highly non-linear traits, reflecting the competitive mechanisms of chemical dispersion, physical packing, and particle lubrication. The rheological behavior of the backfill slurry adheres to the Bingham fluid model (illustrated in Figure 3). This model assumes that the material behaves as a rigid solid until the applied shear stress exceeds a specific yield stress threshold; once this threshold is surpassed, the slurry initiates flow, demonstrating a linear relationship between the shear stress and the subsequent shear rate.

The Bingham rheological model was employed to further analyze the data, which is formulated as follows:

(1) $$\tau =\tau_{0}+\mu_{p}\gamma$$

where *τ* is the shear stress (Pa), τ_0_ is the yield stress (Pa), *μ_p_* is the plastic viscosity (Pa·s), and *γ* is the shear rate (s^−1^).

Figure 3 presents the typical rheological curves illustrating the relationships between shear stress and shear rate, and between the apparent viscosity and shear time. As shown in Figure 3a, the shear stress exhibits an approximately linear relationship with the increasing shear rate, conforming to the characteristic behavior of Bingham fluid. Therefore, the experimental data were fitted to the Bingham rheological model using the least-squares method, determining the corresponding plastic viscosity and yield stress. In Figure 3b, under continuous shearing, the three-dimensional flocculated network of the slurry is progressively disrupted, causing the apparent viscosity to decay rapidly and eventually plateau. This behavior is characteristic of shear-thinning (pseudoplastic) fluids, where the rate of structural breakdown exceeds that of structural reconstruction.

Table 5 shows the rheological properties (yield stress and plastic viscosity) of the filling slurry under different proportions and the Bingham regression formula. The experimental results show that, compared with different variable groups, the rheological parameters of the slurry are highly sensitive to changes in composition; in particular, an increase in polycarboxylate content can greatly reduce the yield stress of the slurry.

### 3.1. Analysis of Slurry Rheological Properties with Different Additives

**Figure 4:**
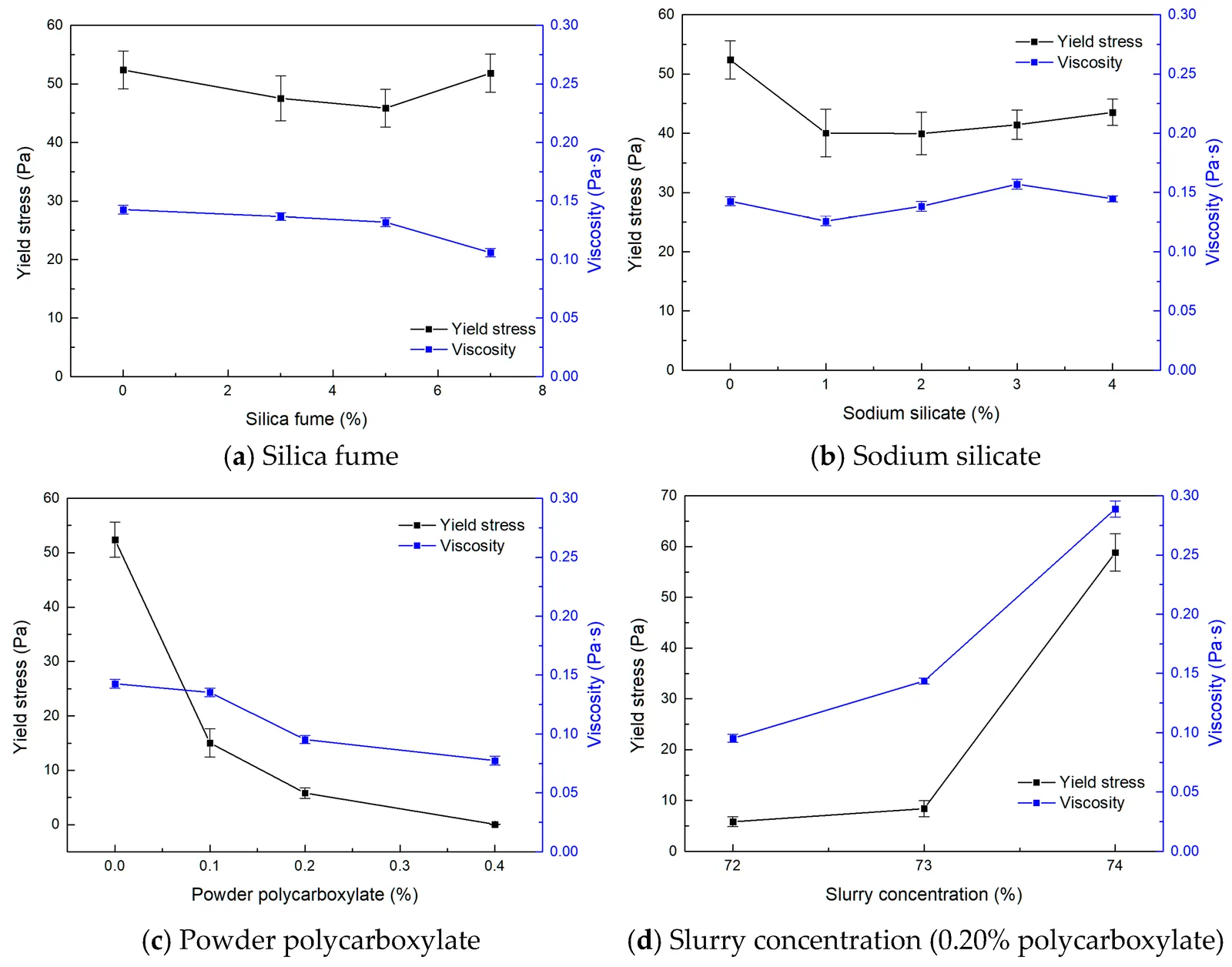
Rheological parameters of backfilling slurry with different additives.

Figure 4 presents the effects of different additives on the rheological parameters of the backfilling slurry. Figure 4a indicates that the influence of silica fume dosage on the rheological parameters of the backfill slurry exhibits a non-linear evolution characteristic. The yield stress of the slurry displays a trend of initially decreasing and subsequently increasing with the increase in silica fume dosage, reaching a minimum of 45.85 ± 3.21 Pa at a dosage of 5% (S3); however, when the dosage further increases to 7% (S4), the yield stress rebounds to 51.82 ± 3.25 Pa. The reason is that silica fume possesses an exceptionally vast specific surface area, where exceeding a threshold dosage causes the excessive microspheres to absorb a significant volume of free water onto their hydrophilic surfaces [23]. Therefore, this reduces the thickness of the excess water lubrication layer coating the coarse tailings, intensifying local particle aggregation and causing the structural yield stress to rebound.

In contrast, the plastic viscosity of the slurry exhibits a distinct monotonic decrease with increasing silica fume content, continuously decreasing from 0.1427 Pa·s at a 0% dosage to 0.1059 Pa·s at 7%. This behavior indicates that incorporating an appropriate dosage of silica fume can effectively reduce the viscous shear resistance between the internal flow layers of the slurry under laboratory rotational shear. This phenomenon demonstrates that the incorporation of an appropriate amount of silica fume effectively reduces the viscous resistance between the internal flow layers of the slurry, thereby improving the flow stability and pumpability of the high-concentration backfill slurry. This validates the capacity of silica fume to optimize the particle size distribution and packing structure of the slurry, which is consistent with the findings of Shao et al. [24]. While reductions in plastic viscosity facilitate flow in pipes, the actual field pumpability is affected by multiple factors, including the wall roughness, pressure gradients, and static bleeding stability. The measured reduction therefore reflects a beneficial decrease in shear resistance within the tested laboratory range, which is plausibly associated with micro-spherical particle lubricating effects rather than a verified optimization of maximum packing fraction in engineering practice.

Figure 4b illustrates that the sodium silicate dosage has a pronounced non-linear effect on both the yield stress and apparent viscosity of the system. As the sodium silicate dosage increases from 0% to 1%, the yield stress decreases markedly. This indicates that introducing a low dosage of sodium silicate weakens the internal structural strength and inter-particle forces within the system, rendering the material more susceptible to flow. Consequently, the viscosity decreases, reflecting a reduction in slurry flow resistance. However, as the sodium silicate dosage further increases to 2–4%, both the yield stress and viscosity experience a certain rebound; in particular, the viscosity reaches a relatively high level around a dosage of approximately 3%. This recovery is attributed to the fact that higher dosages of sodium silicate can enhance the alkalinity of the solution or promote the formation of gel structures and networks, thereby improving the structural integrity and cohesiveness of the system [25].

As shown in Figure 4c, the polycarboxylate dosage has a substantial reducing effect on both the yield stress and apparent viscosity of the system, exhibiting a monotonic decreasing trend with increasing dosage. When the polycarboxylate content increases from 0% to 0.1%, the yield stress declines sharply, indicating that even a small amount of polycarboxylate can significantly weaken the internal structural networks and inter-particle flocculation within the system. Meanwhile, the viscosity of the slurry decreases noticeably, demonstrating an effective improvement in slurry fluidity. With a further increase in the polycarboxylate dosage to 0.2% and above, the yield stress approaches zero, and the system virtually loses its initial shear resistance, implying that the inter-particle forces have been highly dispersed. The viscosity continues to decrease slowly. This suggests that under high-dosage conditions, polycarboxylate primarily maintains the dispersion state through steric hindrance and electrostatic repulsion, while further mitigation of the flow resistance becomes limited [19,26]. This behavior is governed by the electro-steric dispersion of polycarboxylate. The adsorption of the anionic copolymer backbones onto the cementitious particles generates electrostatic repulsion, while the grafted side chains offer steric hindrance to break up particle flocculation [27]. This dispersion releases entrapped water to lubricate the suspension. Accordingly, polycarboxylate exhibits potent dispersing and drag-reducing efficiency, with a highly prominent impact on reducing the yield stress, thereby substantially improving the rheology of the backfill slurries.

### 3.2. Concentration-Driven Rheological Stiffening

The solid mass concentration plays a fundamental role in defining the macroscopic transportability and flow state of CCTB slurries under laboratory conditions. When the mass concentration increases from 72% to 74% (with polycarboxylate maintained at 0.20%), the slurry yield stress surges from 5.82 ± 0.96 Pa to 58.83 ± 3.71 Pa, and the plastic viscosity increases from 0.0953 ± 0.0033 Pa·s to 0.2889 ± 0.0068 Pa·s, as shown in Figure 4d. This non-linear rheological stiffening indicates a potential jamming transition within the multi-phase suspension, although confirming an absolute transition requires independent pipe-loop validation and maximum packing fraction measurements. Under the current laboratory testing conditions, these results define the operating range, where a slight increase in solids content results in a significant decrease in rheological properties.

At a lower solid fraction (72%), aggregate particles remain separated by fluid lubrication layers, permitting easy sliding under minimal shear. Elevating the concentration to 74% is inferred to force the solid volume fraction close to its critical packing limit, severely reducing inter-particle clearance. The local water films rupture, causing a transition from hydrodynamic lubrication to a friction-dominated regime governed by direct contact and inter-particle force chains. Under external shear, these interlocking particle networks demand a significantly higher initial shear stress to disrupt the structural confinement and initiate flow [28].

## 4. Uniaxial Compressive Strength Evolution and Macroscopic Fracture Patterns of Backfill

### 4.1. Macroscopic Mechanical Responses

The macroscopic performance of CCTB specimens across the 13 multi-phase matrices exhibits a highly distinct non-linear evolutionary path over the curing lifecycle. For the micro-silica modified series (S1 to S4), a monotonic mechanical gain is achieved across all milestones (3 d, 7 d, 28 d, and 56 d). The sodium silicate modified series (S5 to S8) acts prominently as an early-stage kinetic accelerator. For the organic polycarboxylate superplasticizer series (S9 to S11), a highly predictable, linear incremental benefit is maintained throughout all curing horizons. When the solid fraction increases from 72% to 74%, the 56-day UCS experiences a high increase compared to the baseline. The detailed specimen UCS information is presented in Table 6.

**Figure 5:**
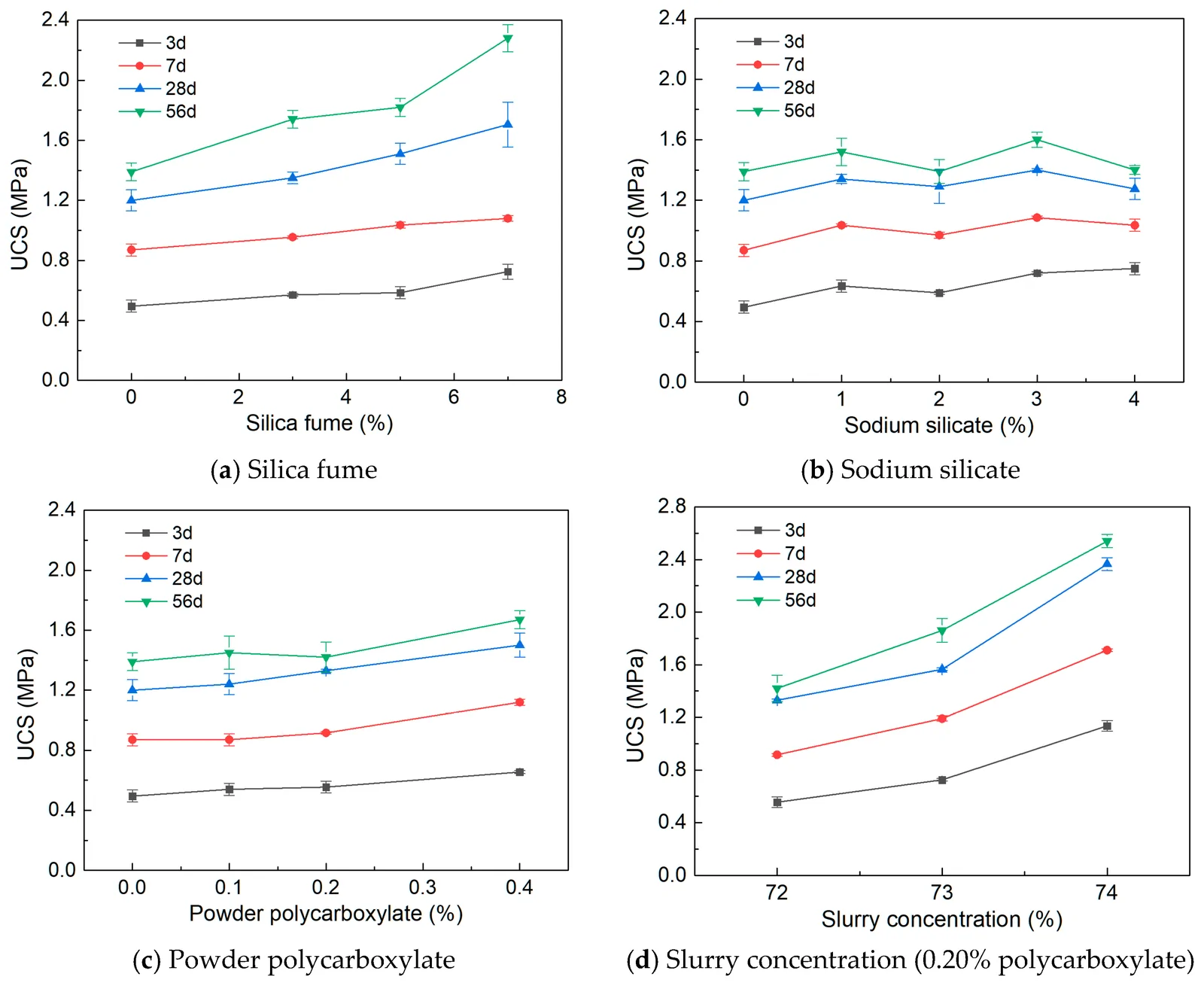
UCS of backfilling specimens with different additive dosages and at different ages.

Figure 5 illustrates the variations in the compressive strength of the cemented specimens at different curing ages as a function of the additive dosage and mass concentration. As shown in Figure 5a, as the silica fume dosage increases stepwise from 0% to 7%, the compressive strength of the backfill at 3, 7, 28, and 56 days exhibits a consistently increasing trend. Specifically, the 56-day compressive strength rises significantly from 1.39 ± 0.06 MPa (S1) to 2.28 ± 0.09 MPa (S4), representing a substantial growth rate of 64%. Based on the established literature, this strengthening is primarily attributed to the physical filling effect of the ultra-fine silica fume particles and the secondary pozzolanic reaction induced by its highly active SiO_2_. These effects significantly improve the microscopic packing density of the paste matrix and generate additional calcium silicate hydrate (C-S-H) gel during hydration, thereby reinforcing the interfacial transition zone (ITZ) and consequently enhancing the load-bearing capacity of the backfill [29].

Figure 5b demonstrates that incorporating sodium silicate exerts a pronounced strengthening effect at early ages. Within the dosage range of 0% to 4%, the backfill strength at all curing ages is enhanced to varying degrees compared to the control group (S1). In particular, the 3-day early strength exhibits a steady upward trend as the dosage increases, rising from 0.50 ± 0.04 MPa to a maximum of 0.75 ± 0.04 MPa. Regarding the long-term strength, the 56-day compressive strength displays two distinct peaks at dosages of 1% (S5) and 3% (S7), reaching 1.52 ± 0.09 MPa and 1.60 ± 0.05 MPa, respectively, but undergoes a slight decline at 2% and 4% dosages. This phenomenon suggests that sodium silicate, acting as an alkaline activator, accelerates the formation of early hydration products as its dosage increases, thereby significantly enhancing the early-age strength. However, an excessive activator dosage may result in an overly rapid hydration rate. Mechanistically, it is hypothesized that this rapid reaction can form a dense coating layer on the particle surfaces that hinders subsequent hydration or lead to an uneven distribution of internal stress, consequently causing the observed fluctuations in the long-term strength development [30]. Similar to the silica fume observations, these passivating barrier effects represent plausible inferred mechanisms rather than directly measured microstructural features.

The results in Figure 5c indicate that increasing the polycarboxylate dosage has a steady positive effect on the strength of the backfill at all curing ages, exhibiting a highly linear growth trend across the whole curing period. As the polycarboxylate dosage increases from 0.10% to 0.40% (S9–S11), the 56-day compressive strength of the backfill progressively rises from 1.45 ± 0.11 MPa to 1.67 ± 0.06 MPa, while the 3-day early-age strength also increases from 0.54 ± 0.04 MPa to 0.66 ± 0.01 MPa. This strength evolution demonstrates that polycarboxylate significantly improves the dispersion and uniformity of solid particles within the slurry, thereby effectively reducing internal defects during the hardening process [31]. Consequently, while substantially decreasing the rheological resistance (as evidenced by the yield stress of S11 dropping to 0.05 Pa), it simultaneously enhances the mechanical properties of the backfill by optimizing its microstructural compactness.

Under a constant polycarboxylate dosage of 0.20%, increasing the solid mass fraction represents the most effective approach to enhancing the mechanical properties of the backfill (Figure 4d). As the overall slurry concentration increases from 72% to 74% (S10, S12, and S13), the backfill strength at all curing ages exhibits a sharp near-linear upward trend. Specifically, the 56-day compressive strength rises from 1.42 ± 0.10 MPa to 2.54 ± 0.05 MPa, representing a substantial increment of 78.9%. This pronounced strengthening effect is attributed to the fact that the increased slurry concentration substantially reduces the internal free water content and porosity, promoting tighter inter-particle contact and thereby facilitating the formation of a more robust skeletal structure upon hardening [32]. However, in conjunction with the rheological characterization of the slurry in Figure 4d, the increased concentration also markedly elevates the rheological resistance; specifically, the yield stress of S13 rises to 58.83 Pa. Consequently, in practical backfilling operations, when aiming to enhance backfill strength by elevating the solid concentration of the slurry, the pumping energy consumption and engineering feasibility must be carefully considered.

### 4.2. Macroscopic Patterns of Specimen Rupture

**Figure 6:**
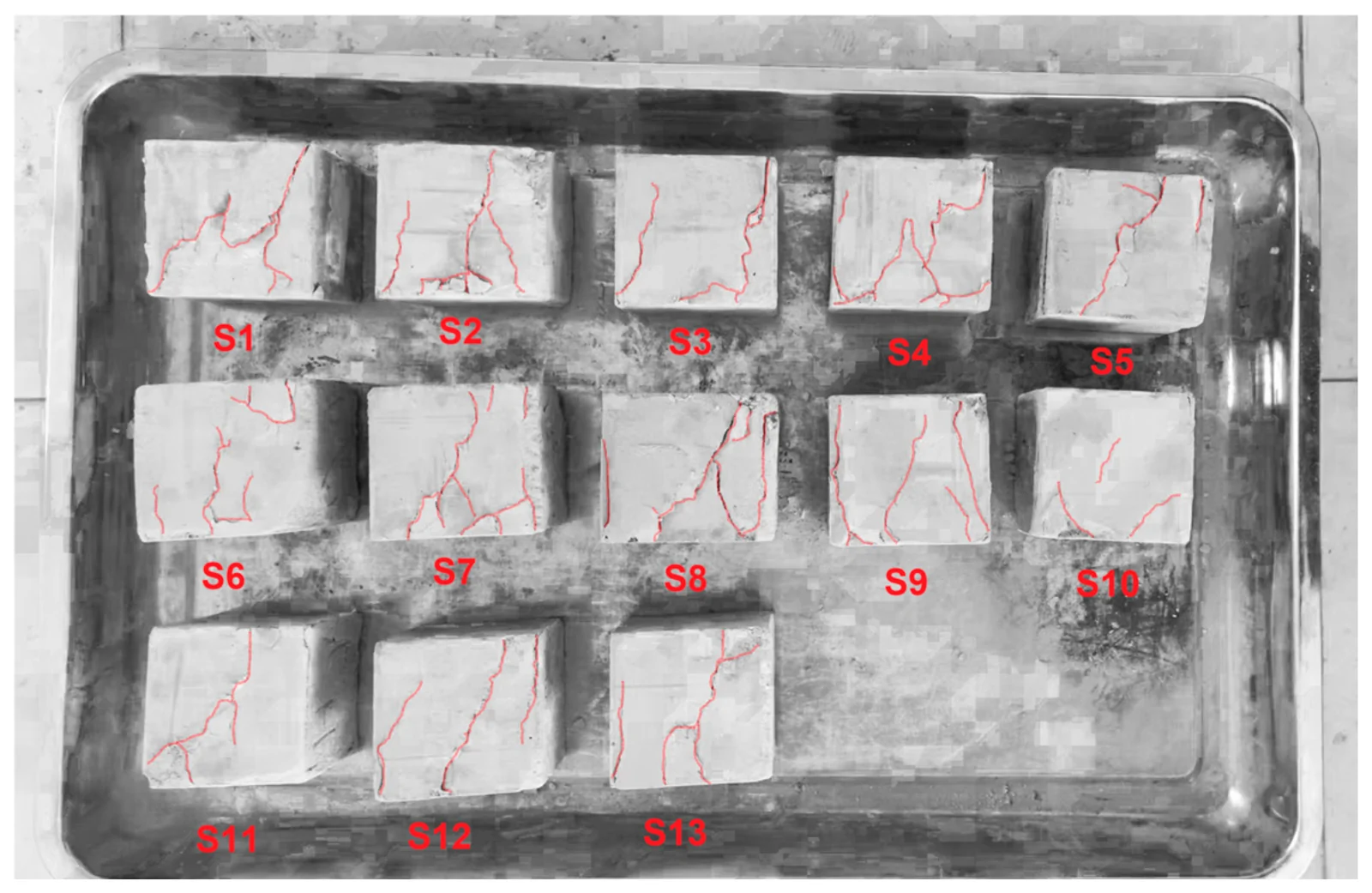
Failure characteristics of the backfilling specimen.

Upon reaching the peak uniaxial load, all CCTB specimens exhibited typical macroscopic brittle failure, as illustrated in Figure 6. The specimen failure was primarily characterized by both longitudinal cracks parallel to the loading direction and oblique shear bands. These macroscopic rupture paths mostly originated at the upper or lower boundary edges and propagated vertically toward the core, appearing as irregular, branched serrations. The number of branches, tortuosity, and width of the main cracks varied among specimens, reflecting the influence of material composition on the microstructure and mechanical properties [32]. The macroscopic fracture patterns varied visibly with the admixture type. The micro-silica series (S1–S4) maintains a highly consistent vertical compression splitting mode. Silica fume can enhance the strength and density of the filling, but it will increase the brittleness of the backfill [33]. Conversely, the sodium silicate series (S5–S8) experienced a structural migration, with fracture patterns shifting from simple isolated macroscopic cracks to highly complex fissure networks. The polycarboxylate series (S9–S11) yielded uniform short fracture lines without anomalous geometric variations. Some specimens (such as S8 and S12) displayed more pronounced oblique shear cracks, though the fundamental failure mechanism, and the layout of the fractured surfaces remained generally unchanged when increasing the solid mass concentration from 72% to 74%.

The specimen failure was characterized by both longitudinal cracks parallel to the loading direction and oblique shear bands. The longitudinal cracks resulted from transverse expansion deformation (the Poisson effect), whereas the oblique fractures indicated localized shear failure. When the transverse tensile stress exceeds the tensile strength of the material, cracks perpendicular to the tensile stress direction (i.e., parallel to the compressive direction) will appear. The cracks mostly start from the edges of the upper and lower surfaces of the specimens and propagate towards the center, with some extending through the entire height of the specimen. Some specimens (such as S8 and S12) showed more obvious oblique shear cracks, indicating shear failure under pressure. All specimens maintained their basic geometric shape after reaching peak failure and did not completely disintegrate, indicating that the specimens have a certain residual strength and integrity.

## 5. Establishment of A Path-Dependent Age-Progressive Strength Prediction Model

The macroscopic strength development of CCTB specimens is a continuous non-equilibrium thermodynamic process driven by prolonged cementitious hydration. Traditional empirical forecasting frameworks treat mechanical strengths at separate curing milestones (such as 3 d, 7 d, 28 d, and 56 d) as isolated static responses, determined solely by initial mix inputs. This assumption mathematically ignores the structural continuity of the material. In reality, the hydration product volume, gel–space ratio, and internal micro-defect topology at a later lifecycle milestone (t) are strictly constrained by the solid frame and microstructural configurations established during the preceding horizon (t-previous).

To capture this physical inheritance, this section introduces an age-progressive coupled mathematical prediction model solved via ordinary least squares regression. By integrating the preceding strength state as a comprehensive status variable, the model is able to capture the continuous trajectory of lifecycle strength evolution, improving the stability and error cancellation under small-sample constraints.

### 5.1. Mathematical Formulation and Dimensionless Normalization

To eliminate scaling discrepancies among different physical quantities and improve the model stability of the regression, a min–max dimensionless normalization is applied to all input variables before regression analysis:

(2) $$x_{i}^{\prime }=\frac{x_{i}-x_{i,min}}{x_{i,max}-x_{i,min}}$$

where $x_{i}$ represents the raw physicochemical design variable (including C, M, S and P); the $x_{i,max}$ and $x_{i,min}$ represent the maximum and minimum operational values within the experimental matrix, respectively. By applying Equation (2), the raw variables are transformed into a uniform [0, 1] scale. Then, the normalized variables such as $C^{\prime },$ $M^{\prime }$, $S^{\prime }$ and $P^{\prime }$ are derived via Equation (2). The specific normalized variables utilized in the model are defined as follows: C′ is the normalized solid mass concentration, M′ is the normalized silica fume dosage, S′ is the normalized sodium silicate dosage, and P′ is the normalized polycarboxylate powder dosage. In order to reflect the continuous path-dependency of hydration kinetics, the mathematical relationship governing the age-progressive strength configuration is defined as follows:

(3) $$f_{c,t}=\alpha_{0}+\alpha_{1}C^{\prime }+\alpha_{2}M^{\prime }+\alpha_{3}S^{\prime }+\alpha_{4}P^{\prime }+\alpha_{5}f_{c,t-previous}$$

where $f_{t}$ denotes the predicted compressive strength at the target curing age (MPa). and t is the number of curing days (3 d, 7 d, 28 d, and 56 d); $f_{c,\;t-previous}$ represents the verified companion compressive strength from the preceding curing age retained directly in its dimensional form (MPa) to maintain physical interpretability; α_0_ is the constant term; and $\alpha_{i}$ (I = 1–5) is the calibrated regression coefficient corresponding to the normalized formulation inputs and the preceding state variable.

### 5.2. Model Calibration and Statistical Correlation Analysis of Regression Parameters

By executing multivariate linear regression on the 13 formulation groups, the age-progressive prediction formulas for each milestone are calibrated as follows (Equations (4)–(7)). The resulting regression coefficients act as a direct statistical correlation index, quantifying the sensitivity of the backfill strength to each specific mix variable across different curing ages.

3-Day Strength Prediction Formula (Baseline Phase):

(4) $$f_{c,3d}=-18.38+19.40C^{\prime }+2.04M^{\prime }+4.92S^{\prime }+25.03P^{\prime }$$

7-Day Strength Prediction Formula (Progressive Phase I):

(5) $$f_{c,7d}=-4.47+4.95C^{\prime }+0.13M^{\prime }-1.02S^{\prime }+7.69P^{\prime }+1.05f_{c,3d}$$

28-Day Strength Prediction Formula (Progressive Phase II):

(6) $$f_{c,28d}=-2.54+2.71C^{\prime }+2.13M^{\prime }-2.13S^{\prime }+2.77P^{\prime }+1.26f_{c,7d}$$

56-Day Strength Prediction Formula (Progressive Phase III):

(7) $$f_{c,56d}=-8.33+9.02C^{\prime }+4.55M^{\prime }-0.34S^{\prime }+4.69P^{\prime }+0.79f_{c,28d}$$

The calibrated regression coefficients and respective determination coefficients, comparing the traditional decoupled static equations against the proposed age-progressive models and respective determination coefficients, are presented in Table 7. Because five predictor terms were fitted to only 13 formulation-level observations at each stage, the calibrated coefficients should be regarded primarily as empirical fitting parameters indicative of general trends, rather than as independent physical sensitivity parameters.

The calibrated parameters (from α_1_ to α_4_) clarify the varying impact of the initial mix design variables across the backfill lifecycle. The slurry mass concentration coefficient (α_1_) remains consistently positive across all milestones, peaking at 19.40 during the 3-day phase. This confirms that the initial slurry concentration plays a major role in early structural development by optimizing the initial skeletal packing and decreasing the initial porosity. Similarly, the polycarboxylate powder coefficient (α_4_) exhibits an exceptionally high value of 25.03 in the 3-day model, far exceeding all other parameters. This highlights the early mechanical benefits of polycarboxylate-induced deflocculation, which creates a highly uniform distribution of hydration products and minimizes structural micro-defects. While the influence of both concentration and polycarboxylate scales down in later stages, they maintain a positive contribution to long-term strength development.

The sensitivity trends for mineral admixtures reveal distinct structural evolution mechanisms. The micro-silica coefficient (α_2_) is consistently positive and increases substantially during the 28-day (α_2_ = 2.13) and 56-day (α_2_ = 4.55) horizons. Conversely, the sodium silicate coefficient (α_3_) changes sign over time, exhibiting a positive correlation at 3 days (α_3_ = 4.92) before turning negative at 7 days (α_3_ = −1.02), 28 days (α_3_ = −2.13) and 56 days (α_3_ = −0.34).

The coefficient (α_5_) is consistently positive across all progressive models, reaching 1.05 at 7 days, 1.26 at 28 days, and 0.79 at 56 days. This positive correlation indicates that the mechanical capacity developed in earlier stages provides a foundational framework for subsequent strength growth. The coefficient peaks in the 28-day model (α_5_ = 1.26), demonstrating that mid-term structural development depends heavily on the quality and integrity of the early skeleton formed during the initial 7-day curing phase.

### 5.3. Evaluation of Predictive Accuracy and Convergence Performance

**Figure 7:**
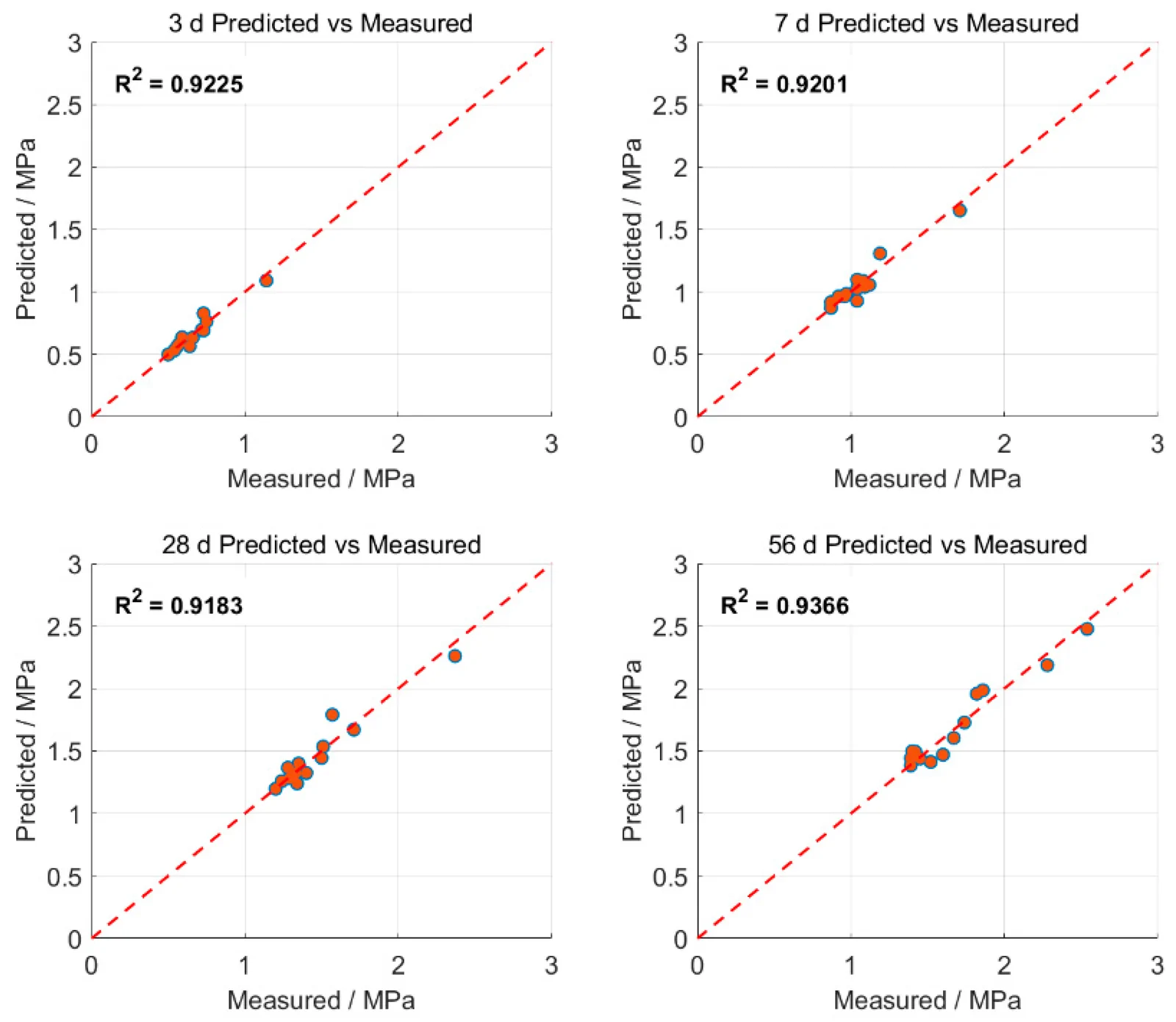
Comparison of predicted and measured strength values (non-progressive model).

**Figure 8:**
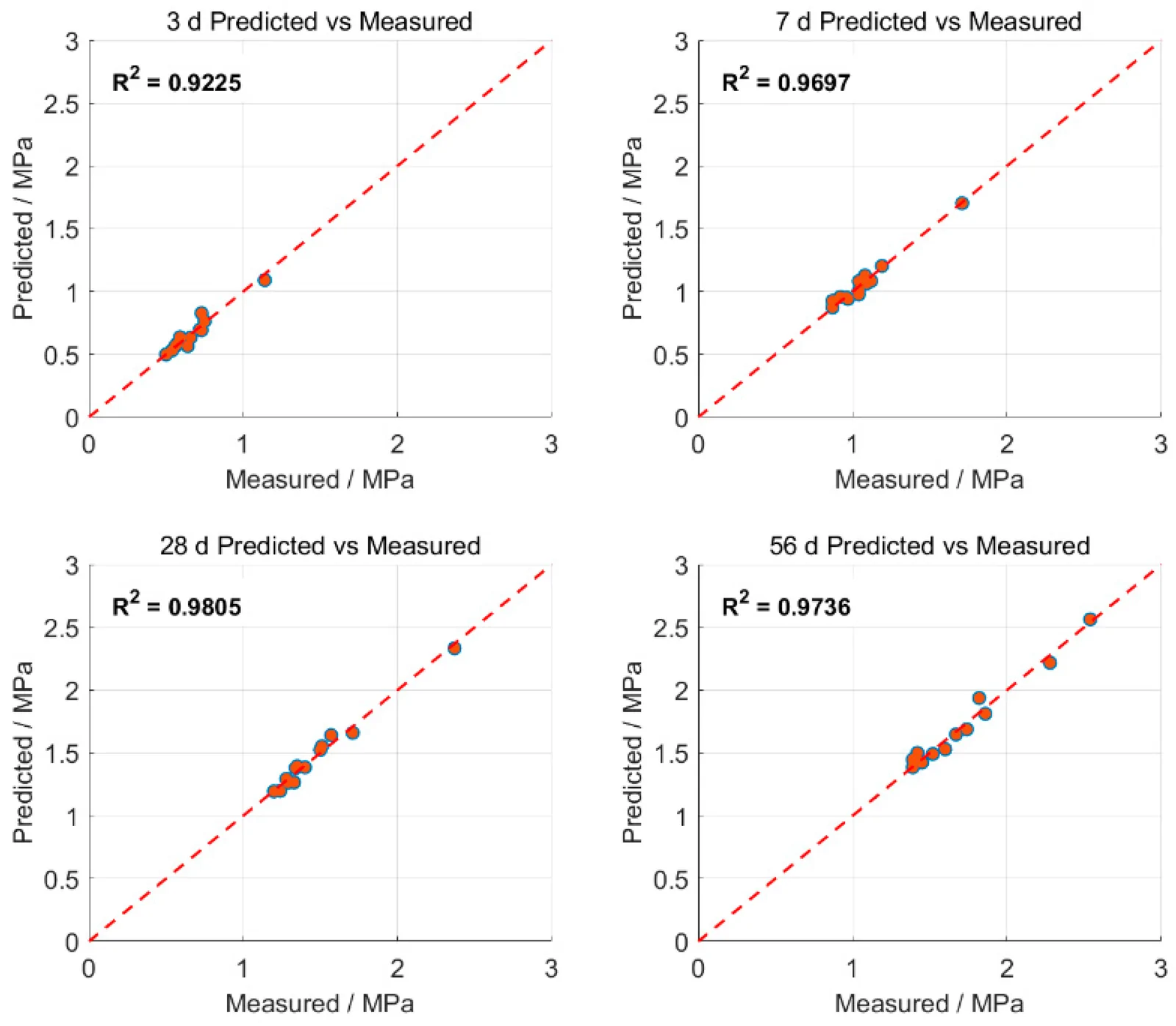
Comparison of predicted and measured strength values (progressive model).

A comparison between the independent static equations and the proposed progressive framework demonstrates the clear mathematical advantages of incorporating path-dependent continuity. When evaluated using independent regression equations, the determination coefficients (R^2^) for the 3-day, 7-day, 28-day, and 56-day milestones are 0.9225, 0.9201, 0.9183, and 0.9366, respectively (as summarised in Table 7 and illustrated in Figure 7). In Figure 7 and Figure 8, the red dots represent the experimental data points, and the red dashed line represents the ideal 1:1 fitting line (y = x), indicating perfect agreement between the predicted and measured values. Although these values demonstrate reasonable baseline correlations, this decoupled architecture fundamentally assumes that strength outputs at distinct ages are mutually independent, thereby completely neglecting the cumulative microstructural inheritance over time.

In contrast, the age-progressive framework established herein introduces a sequential chain-linked architecture. By incorporating the verified mechanical strength of the preceding age (*f_c,t-previous_*) into the subsequent milestone equation, the model transitions from a static boundary value problem to a continuous path-dependent framework. By transitioning to the age-progressive framework, the prediction accuracy improves significantly. As shown in Figure 8, the progressive models achieve higher determination coefficients, yielding an R^2^ of 0.9697 at 7 days, 0.9805 at 28 days, and 0.9736 at 56 days. Notably, the 28-day strength prediction accuracy experiences a substantial improvement, with the R^2^ value rising from 0.9183 to 0.9805.

From the perspective of stochastic processes, this progressive model captures the sequential evolution of the structural states, where the structural state at time *t* is fully defined by the state at the previous time step and the current hydration variables. By treating the preceding strength *f_c,t-previous_* as a holistic indicator of early hydration degrees and internal defect distributions, the progressive model inherently suppresses random data noise. This promotes reliable mathematical convergence even under small-sample constraints.

To validate the out-of-sample predictive power of the age-progressive model, a leave-one-out cross-validation (LOOCV) protocol was implemented. By systematically isolating a single formulation group (e.g., removing S1) for testing while training the regression coefficients on the remaining other twelve groups, the model was forced to predict unseen data. When utilizing the experimentally measured prior-age UCS as the input variable, the LOOCV generated R^2^ values ranging from 0.7951 to 0.8198 for the 7-day to 56-day milestones. As this procedure highly relies on the measured prior-age strength, it strictly functions as a conditional updating test rather than an independent recursive forecast based solely on the initial mix design. Considering the limited sample size of 13 formulation groups, there is substantial uncertainty in the model performance, and these preliminary LOOCV results cannot definitively validate the generalized predictive reliability across broader conditions. Instead, consistent with the limitations of the dataset, these findings indicate that the path-dependent framework has the potential to serve as a practical field-updating tool to adjust mid-to-late-stage expectations once early-age testing data becomes available.

## 6. Conclusions

Based on the experimental investigation and theoretical analysis of the rheological properties, compressive damage evolution, and strength prediction of CCTB, the main conclusions are drawn as follows:

(1) Polycarboxylate powder proved to be the most effective rheology modifier within the tested range. At a dosage of 0.40%, it reduced the yield stress to 0.05 Pa and the plastic viscosity to 0.0775 Pa·s. However, stability against bleeding and segregation should be verified prior to field application.
(2) Increasing the silica fume content from 0% to 7% increased the 56 d UCS by 64.0%. Sodium silicate effectively enhanced the early-age strength but exhibited non-monotonic effects on the 28 and 56 d strength. These mechanisms remain inferential without direct microstructural measurements.
(3) Increasing the solids concentration from 72% to 74% raised the 56 d UCS from 1.42 to 2.54 MPa but also raised the yield stress from 5.82 to 58.83 Pa, demonstrating a clear transport-strength trade-off rather than a single optimum.
(4) The proposed age-progressive regression improves conditional prediction when a measured prior-age UCS is available. It does not yet demonstrate superior recursive prediction for unseen mixtures, and independent validation with a larger factorial dataset is required.

The results identify a pronounced transport–strength trade-off. Higher solids concentration was the most effective strength-enhancement route in the tested concentration series, but it also caused the largest increase in rheological resistance. Polycarboxylate powder reduced the fitted yield stress and partly offset this resistance, although very low yield stress may compromise static stability. A field mix design should therefore define simultaneous acceptance criteria for the pump pressure, bleeding, setting time, and required UCS, rather than focusing solely on maximizing the UCS.

The conclusions are limited to the specific tailings source, CKD batch, and the fixed binder formulation (10% CKD, a binder-to-tailings ratio of 1:6), and the single-factor matrix does not quantify interactions among admixtures. Mechanistic interpretations were inferred from macroscopic behavior and lack microstructural validation. Therefore, future research will priorities advanced microstructural investigations, utilizing scanning electron microscopy (SEM) and X-ray diffraction (XRD), to explicitly reveal the hydration products, gel formations, and phase assemblages of CKD-modified backfill, which govern the observed transport-strength trade-off. In addition, the rheological tests characterize shear resistance under controlled laboratory conditions, and their application to pipeline transportability, critical packing limits, or pumping pressure losses requires further validation through pipe-loop testing, pressure measurements, and stability assessments. The regression dataset contains only 13 averaged observations and no independent external validation set; so, the generalization to broader conditions or multi-component systems requires further experimental verification.

## Figures and Tables

**Table 1:** Chemical composition of mine tailings.

Ingredient	SiO_2_	Al_2_O_3_	Na_2_O	K_2_O	Fe_2_O_3_	CaO	MgO	MnO	TiO_2_	SO_3_	LOI *
Percentage/%	46.45	2.42	0.16	0.34	1.29	12.98	11.57	0.10	0.19	0.48	23.90

* LOI (Loss on ignition).

**Table 2:** Chemical composition of OPC.

Ingredient	Al_2_O_3_	Fe_2_O_3_	K_2_O	Na_2_O	SiO_2_	CaO	MgO	SO_3_	Free CaO	LOI *
Percentage/%	5.24	3.15	0.55	0.21	20.92	61.85	2.10	2.58	0.85	2.25

* LOI (Loss on ignition).

**Table 3:** Chemical composition of CKD.

Ingredient	Al_2_O_3_	Fe_2_O_3_	K_2_O	Na_2_O	SiO_2_	CaO	MgO	SO_3_	Free CaO	LOI *
Percentage/%	3.81	1.78	0.50	0.54	8.58	58.10	3.60	2.88	10.72	9.41

* LOI (Loss on ignition).

**Table 4:** Test program.

Specimen ID	Slurry Concentration (C)	Silica Fume (M)	Sodium Silicate (S)	Polycarboxylate Powder (P)
S1	72%	0%	0%	0%
S2	72%	3%	0%	0%
S3	72%	5%	0%	0%
S4	72%	7%	0%	0%
S5	72%	0%	1%	0%
S6	72%	0%	2%	0%
S7	72%	0%	3%	0%
S8	72%	0%	4%	0%
S9	72%	0%	0%	0.10%
S10	72%	0%	0%	0.20%
S11	72%	0%	0%	0.40%
S12	73%	0%	0%	0.20%
S13	74%	0%	0%	0.20%

**Table 5:** Rheological parameters of backfill slurry.

Specimen ID	Yield Stress (Pa)	Plastic Viscosity (Pa·s)	Bingham Regression Equation
S1	52.38 ± 3.24	0.1427 ± 0.0037	τ = 52.38 + 0.1427γ
S2	47.52 ± 3.80	0.1367 ± 0.0033	τ = 47.52 + 0.1367γ
S3	45.85 ± 3.21	0.1319 ± 0.0038	τ = 45.85 + 0.1319γ
S4	51.82 ± 3.25	0.1059 ± 0.0037	τ = 51.82 + 0.1059γ
S5	40.03 ± 4.02	0.1259 ± 0.0040	τ = 40.03 + 0.1259γ
S6	39.95 ± 3.59	0.1384 ± 0.0041	τ = 39.95 + 0.1384γ
S7	41.42 ± 2.49	0.1570 ± 0.0040	τ = 41.42 + 0.1570γ
S8	43.52 ± 2.23	0.1447 ± 0.0025	τ = 43.52 + 0.1447γ
S9	15.03 ± 2.57	0.1353 ± 0.0035	τ = 15.03 + 0.1353γ
S10	5.82 ± 0.96	0.0953 ± 0.0033	τ = 5.82 + 0.0953γ
S11	0.05 ± 0.04	0.0775 ± 0.0038	τ = 0.05 + 0.0775γ
S12	8.41 ± 1.58	0.1436 ± 0.0026	τ = 8.41 + 0.1436γ
S13	58.83 ± 3.71	0.2889 ± 0.0068	τ = 58.83 + 0.2889γ

**Table 6:** UCS of backfill specimens at different curing ages.

Specimen ID	3 d UCS (MPa)	7 d UCS (MPa)	28 d UCS(MPa)	56 d UCS (MPa)
S1	0.50 ± 0.04	0.87 ± 0.04	1.20 ± 0.07	1.39 ± 0.06
S2	0.57 ± 0.01	0.96 ± 0.01	1.35 ± 0.04	1.74 ± 0.06
S3	0.59 ± 0.04	1.04 ± 0.02	1.51 ± 0.07	1.82 ± 0.06
S4	0.73 ± 0.05	1.08 ± 0.02	1.71 ± 0.15	2.28 ± 0.09
S5	0.64 ± 0.04	1.04 ± 0.01	1.34 ± 0.03	1.52 ± 0.09
S6	0.59 ± 0.01	0.97 ± 0.02	1.29 ± 0.11	1.39 ± 0.08
S7	0.72 ± 0.01	1.09 ± 0.01	1.40 ± 0.01	1.60 ± 0.05
S8	0.75 ± 0.04	1.04 ± 0.04	1.28 ± 0.07	1.40 ± 0.03
S9	0.54 ± 0.04	0.87 ± 0.04	1.24 ± 0.07	1.45 ± 0.11
S10	0.56 ± 0.04	0.92 ± 0.01	1.33 ± 0.01	1.42 ± 0.10
S11	0.66 ± 0.01	1.12 ± 0.02	1.50 ± 0.08	1.67 ± 0.06
S12	0.73 ± 0.01	1.19 ± 0.02	1.57 ± 0.01	1.86 ± 0.09
S13	1.14 ± 0.04	1.71 ± 0.01	2.37 ± 0.05	2.54 ± 0.05

**Table 7:** Calibrated regression parameters for multi-age strength prediction models.

Regression Coefficient	3 d Model	7 d Non-Progressive	7 d Progressive	28 d Non-Progressive	28 d Progressive	56 d Non-Progressive	56 d Progressive
α_0_ (Constant)	−18.38	−23.86	−4.47	−32.60	−2.54	−34.00	−8.33
α_1_ (C′)	19.40	25.41	4.95	34.74	2.71	36.37	9.02
α_2_ (M′)	2.04	2.28	0.13	5.00	2.13	8.48	4.55
α_3_ (S′)	4.92	4.16	−1.02	3.12	−2.13	2.12	−0.34
α_4_ (P′)	25.03	34.08	7.69	45.72	2.77	40.69	4.69
α_5_ (Preceding UCS f_c,t-previous_)	-	-	1.05	-	1.26	-	0.79
R^2^ (determination coefficients)	0.9225	0.9201	0.9697	0.9183	0.9805	0.9366	0.9736

## Data Availability

The original contributions presented in this study are included in the article. Further inquiries can be directed to the corresponding author.
